# Assessing Law No. 87 of 2024 on private sector management of public healthcare facilities in Egypt: neoliberal roots, potential consequences, and policy recommendations

**DOI:** 10.1186/s12913-026-14348-x

**Published:** 2026-04-02

**Authors:** Wafa Abu El Kheir-Mataria, Hala Abou-Taleb, Sameh El-Saharty

**Affiliations:** 1 Institute of Global Health and Human Ecology, The American University in Cairo, Cairo, Egypt; 2 Independent Global Public Health Professional, Cairo, Egypt; 3 Senior Health Policy Advisor, Former Lead Health Policy Specialist, The World Bank, Washington, DC USA

**Keywords:** Egypt, Health governance, Neoliberalism, Health equity, Private sector, Health policy reform, Law No. 87

## Abstract

**Background:**

In March 2024, Egypt enacted Law No. 87, permitting private sector management of public healthcare facilities. The timing of the law reflects the intersection of domestic challenges including fiscal pressure, institutional capacity constraints, and limitations in public health service delivery, and global policy influences, including neoliberal approaches to economic development. This study analyses the law’s underlying drivers and assesses its anticipated implications for equity, access, and health system governance.

**Methods:**

A critical political economy of health framework guided this qualitative study. We conducted a multi-level policy analysis of Law No. 87 and relevant regulatory texts, supported by a desk review of scholarly and grey literature. To enhance contextual depth and triangulate findings, we convened a multidisciplinary roundtable with 14 experts from government, academia, civil society, and development agencies. Transcripts were thematically analysed.

**Results:**

Findings indicate that Law No. 87 reflects a broader policy trajectory of the adoption of neoliberal economic development, which in the health sector, signals a major health policy shift. For the first time, the private sector is provided a legal framework to manage public health facilities. While some experts viewed it as a necessary, efficiency-oriented reform that would improve quality of health services, others flagged concerns over vague regulatory provisions, limited public oversight, and weak implementation capacity. The absence of community participation and explicit equity safeguards raised further concerns about the law’s ability to advance inclusive and accountable health system governance.

**Conclusion:**

Law No. 87 signals a major shift in the governance of public healthcare in Egypt. Beyond efficiency goals, its long-term impact will depend on the state’s capacity to regulate, monitor, and steer implementation toward equity objectives. Addressing gaps in accountability, ensuring stakeholder participation, and articulating clear equity safeguards will be essential for aligning the reform with Egypt’s commitments to universal health coverage and protecting the public interest.

## Introduction

In 2024, Egypt passed Law No. 87, which grants private entities the right to manage, operate, and develop public healthcare facilities. This marks a major progression in health policy, positioning private sector actors within the core of the public health delivery system [1]. The law includes clauses on contract duration, workforce retention, public service requirements, and crisis response obligations. As Egypt’s healthcare system undergoes significant changes in governance and delivery models, it becomes essential to situate this reform within the broader historical and ideological context that enabled its emergence.

Egypt’s health system is marked by a large but under-resourced public sector that coexists with a rapidly expanding private sector, resulting in fragmented service delivery, high out-of-pocket spending, and persistent inequities. Fiscal constraints, workforce shortages, and the slow and uneven rollout of the Universal Health Insurance Law have intensified pressure to introduce new management models for public facilities. Within this context, Parliament rapidly advanced and approved Law No. 87 in March 2024, framing it as a mechanism to improve efficiency, attract private investment, and activate underutilized infrastructure. The law authorizes Egyptian and foreign private entities to manage, operate, and rehabilitate public healthcare facilities through concession agreements, while maintaining services for public beneficiaries and retaining a portion of the workforce. These provisions represent a significant shift in the governance of public healthcare delivery and underscore the need to assess the equity, accountability, and system-wide implications of this reform.

The growing involvement of the private sector in public services has long been associated with neoliberal ideology. Neoliberalism is commonly understood as the economic model that replaced Keynesianism. It prioritizes market-based solutions, privatization, and a limited role for direct state intervention. Beyond a narrow economic doctrine, neoliberalism has also been described as a “hegemonic paradigm” that operates at a deeper level, shaping policy agendas and social discourse, and influencing contemporary policy trends, including calls for the liberalization and privatization of healthcare systems [2].

Neoliberalism fundamentally frames health as an individual responsibility rather than a public good. It reduces state involvement in public services, shifting attention away from collective health goals and diminishing the state’s role in addressing public health needs. Moreover, neoliberalism, as a market-centric approach that prioritizes consumer-oriented service delivery, presents structural challenges for health systems [3]. Neoliberal health reforms are commonly justified through claims of cost-effectiveness and efficiency, often deprioritizing equity particularly in contexts marked by pre-existing social and economic inequalities. These reforms may exacerbate disparities by limiting access to essential health services, sidelining health needs of low-income and vulnerable populations, and reshaping healthcare delivery in ways that disproportionately benefit wealthier groups [4, 5]. As such, health equity becomes a critical lens through which to examine the implications of neoliberal health reforms. Health equity, defined as the absence of avoidable and unjust differences in health outcomes [6], requires attention to the structural determinants of service delivery, financing, and access, all of which are shaped by market-oriented policy frameworks.

This study investigates how historical, structural, and ideological forces including but not limited to neoliberalism have shaped Egypt’s recent health policy trajectory. It explores the economic and institutional drivers behind Law No. 87 and critically examines its potential implications for equity, access, and public accountability, particularly for marginalized populations. The study also identifies policy options to mitigate risks and align implementation with public health and social justice goals. By situating the reform within Egypt’s evolving health system, it offers timely insights on balancing efficiency-oriented reforms with equity-driven governance.

## Methods

This study employs a qualitative approach to critically analyse Egypt’s 2024 Law No. 87 on the Private Sector Management of Public Healthcare Facilities. It is an exploratory design based on a critical political economy of health approach, with a strong focus on health equity. It aims to understand how broader economic and political factors, including power dynamics and institutional structures, influence health policy decisions. The research integrates a document-based policy analysis with empirical insights derived from a round table discussion with Egyptian health policy experts. The study takes an interpretive perspective, allowing expert insights to shape the analysis and highlight different viewpoints on the law and its potential impact.

A critical political economy of health approach examines how power relations, institutional arrangements, and dominant economic ideologies shape health policies and health system outcomes, with particular attention to their implications for equity and access [2, 7]. This approach is well established in global health and health policy scholarship and has been widely used to analyse neoliberal reforms, privatization, and the reconfiguration of state responsibility in low- and middle-income countries. Consistent with this tradition, the present study combines analysis of legal texts with a review of academic and grey literature and qualitative expert perspectives to interpret how Law No. 87 reflects broader political-economic forces and how these forces may influence governance, equity, and accountability in the health system. This integrative and interpretive use of multiple data sources aligns with established political economy methodologies in health policy analysis.

### Research questions

1. How have decades of neoliberal policies influenced the development of the 2024 law on private sector management of public healthcare facilities in Egypt?
2. What are the economic and healthcare challenges that necessitated the introduction of this law?
3. What potential risks and benefits does the law pose for health equity and access to healthcare services in Egypt?
4. What strategies can be implemented to mitigate potential adverse effects of this law on public healthcare?

### Participants and expert selection

Twenty invitations were initially sent by email to individuals with recognized expertise in healthcare governance, service delivery, and social justice in Egypt. Fourteen experts ultimately participated in the session. Participants came from diverse institutional backgrounds, including academic institutions involved in health systems research and public policy, Egyptian NGOs working on health rights and community-based service provision, health equity advocates with experience in underserved areas, rights-based civil society organizations focused on access to care, international and multilateral development organizations, and the Ministry of Health and Population. This diversity ensured a rich, multi-perspective discussion that reflected key institutional viewpoints.

Participants were selected based on recognized professional expertise in healthcare governance and policy. Some participants were known to the research team through professional networks; however, no prior research relationship influenced data collection or interpretation.

The roundtable was held in Cairo on December 18, 2024 and lasted approximately two hours. The session was facilitated by the lead author (W.A.M.), PhD in Health Policy and Management and Senior Researcher at the Institute of Global Health and Human Ecology, American University in Cairo. The facilitator has prior experience conducting qualitative policy research and moderating expert discussions in health systems governance. Four observers were present during the session in a non-participatory capacity. The discussion was conducted in Arabic using a semi-structured guide developed from the study’s research questions. The session was audio recorded, and the facilitator also took field notes to capture contextual observations and non-verbal dynamics.

### Ethical considerations

This study received ethical approval from the Institutional Review Board at the American University in Cairo (AUC), approval number CASE #2024-2025-059. All roundtable participants were provided with a written informed consent form outlining the study’s purpose, procedures, and confidentiality measures. Participation was voluntary, and consent was obtained prior to data collection. Confidentiality was ensured unless participants explicitly requested to be acknowledged by name.

### Data analysis

The analysis consisted of two interconnected components. First, a document-based policy analysis was conducted to examine the structure and framing of relevant legal and policy documents, including Law No. 87 of 2024, and its executive regulations (Prime Ministerial Decree No. 2851/2024). These texts were reviewed to identify provisions related to privatization, equity, accountability, and the role of the state in health service delivery. Relevant academic and gray literature was also consulted to support interpretation and contextualize the policy changes within Egypt’s broader neoliberal reform trajectory.

Second, the transcript of the expert round table discussion was analysed using thematic analysis. The transcript was read multiple times for familiarization, and initial codes were generated inductively. Coding was conducted manually by the lead author using Microsoft Word. Codes were then organized into categories and developed into broader themes reflecting participants’ views on the law’s rationale, potential equity implications, implementation challenges, and systemic risks. Emerging themes were subsequently reviewed and refined collaboratively with co-authors to enhance analytical rigor and ensure coherence with the broader political economy framework. Transcripts were shared with participants for comment and clarification to enhance accuracy and credibility. Round table thematic findings were triangulated with insights from the policy document analysis to identify areas of convergence, divergence, and emphasis, thereby strengthening the credibility and depth of interpretation.

### Limitations

Although the study offers in-depth insights into the political economy and equity dimensions of Law No. 87, it has limitations. The roundtable was conducted as a single-session event, which may have constrained the depth and diversity of perspectives compared to multi-session or longitudinal engagements. Given this exploratory design, formal thematic saturation was not assessed. Additionally, expert selection was based on purposive sampling. Although efforts were made to ensure institutional diversity, the views expressed may not fully represent all stakeholder groups, particularly frontline healthcare workers or patients. While credibility was strengthened through triangulation of roundtable findings with legal document analysis and relevant literature, the interpretation of findings is shaped by the study’s critical political economy framework, which emphasizes structural power dynamics and equity considerations. Alternative analytical approaches may have yielded different emphases. Finally, as the law’s implementation is still at an early stage, the analysis is based on anticipated rather than observed impacts, which may evolve over time. Further research is needed as the law is rolled out.

## Results

### Contextual analysis and policy background

#### The neoliberal foundations of Law 87

Neoliberalism in Egypt emerged through a combination of domestic and international pressures, beginning in the 1970s and intensifying in the 1990s with structural adjustment programs. The “Infitah” (open-door) policy marked a shift from the socialist economic policies. Infitah policy opened Egypt’s economy to foreign investment, reduced state control over certain sectors, and promoted private enterprise, laying the groundwork for neoliberal reforms [8]. The most significant push came in the 1990s, when Egypt faced mounting macroeconomic pressures, including balance-of-payments constraints, rising external debt, and fiscal stress.

These conditions prompted engagement with the International Monetary Fund (IMF) and the World Bank. Both called for structural adjustment programs, leading to the introduction of large-scale privatization of state-owned enterprises, deregulation, reduced public spending, and reforms to integrate Egypt into the global market [8]. Neoliberal economic policies such as privatization and austerity have reinforced socio-economic inequities. The state’s neoliberal orientation prioritized elite and politically connected individuals’ interests. Policies exacerbated public resource scarcity and limited access to essential services. Elite and politically connected individuals gained control over key industries like real estate, finance, and manufacturing.

While neoliberal reforms reshaped labour markets, public spending, and social protection in ways that increased vulnerability for lower-income groups, the concentration of economic power among politically connected elites further mediated these effects, intensifying inequality and limiting the redistributive capacity of the state.

This led to increased wealth concentration and created a class with vested interests in maintaining the status quo, even as poverty and inequality persisted or worsened for much of the population, undermining social welfare [9]. Poverty rates reflected this deterioration: CAPMAS reported an increase from 21.6% in 2008/2009 to 26.3% in 2012/2013, and World Bank estimates placed it at 27.8% by 2015, signalling the deepening of hardship for large segments of the population during the neoliberal restructuring [10, 11].

Moreover, the Egyptian state’s neoliberal strategy has prioritized sectors like tourism and real estate over essential public investments, limiting job creation and weakening economic resilience. The neoliberal logic that favours profitability and rapid financial returns over industrial development and public service provision, has led to imbalanced economic outcomes and heightened vulnerabilities within Egypt’s economy [12]. As neoliberal policies continued post-2011, international institutions and trading partners shaped Egypt’s reform trajectory through financing arrangements, policy conditionalities, and technical assistance that reinforced commitments to an open-market economy. This dynamic highlights how neoliberalism became embedded in Egypt’s governance, persisting as the guiding framework amid political upheaval [8].

Diminished social welfare and economic instability have fuelled widespread public discontent. The gains from economic reforms were unevenly shared, leaving vulnerable populations to face the greatest impact from decreased state support in essential public sectors [13].

#### Egypt’s health system and neoliberalism

The Egyptian state’s shift towards a neoliberal welfare model following economic adjustment policies minimized the state’s role in social welfare, prioritizing control over service provision and shifting responsibility to the private sector. This approach formed part of a broader policy trajectory associated with neoliberal reform agendas that reduced public investment in welfare, including healthcare. According to the World Bank (2022), Egypt’s public spending on health accounted for only 1.8% of GDP and 7.2% of total government expenditure, with per capita public health spending at just $64.8. At the same time, out-of-pocket payments made up 53.8% of total health expenditures, indicating a heavy reliance on household spending and limited state support [14]. This reflects a shift away from universal service provision toward selective, limited support aimed primarily at maintaining social order rather than ensuring equitable access for all [15].

Neoliberal economic reforms have left a lasting imprint on Egypt’s health sector, reinforcing market-oriented policies and reduced public investment. Since the early 1990s, structural adjustment and fiscal austerity have curtailed state spending on healthcare, expanding reliance on private providers and creating a fragmented, tiered system. Wealthier groups access high-quality private care, while lower-income populations face under-resourced public facilities. By 2008, 72% of total health expenditure came from private sources, mainly out-of-pocket, highlighting the financial burden shifted onto households. Privatization efforts, often guided by profitability rather than public need, have compromised universal access and exacerbated health disparities across social strata [16].

Inadequate healthcare coverage in Egypt exposes vulnerable populations to catastrophic health expenditures. The country’s regressive financing structure places a disproportionate burden on lower-income groups, primarily due to heavy reliance on out-of-pocket payments [17]. In 2020, such payments accounted for 59.31% of current health expenditure [18]. These costs have pushed many households below the poverty line, often forcing families to deplete savings or take on debt to access care, highlighting the urgent need for stronger public health financing models to protect against financial hardship [19].

Neoliberal policies emphasizing fiscal constraints and private sector reliance have produced a fragmented, tiered healthcare system that exacerbates inequities [20]. In this structure, access and quality are stratified by socioeconomic status: wealthier individuals benefit from faster, higher-quality care through private channels, while lower-income groups rely on overcrowded, under-resourced public facilities, often facing delayed or inadequate treatment [21].

Social justice in healthcare is ensuring fair access and quality across all groups, with the aim of reducing disparities rooted in socioeconomic inequalities, not only economically but also geographically. Egypt’s neoliberal reforms and reduced investment in welfare resulted in uneven resource distribution, exacerbating health inequalities in rural areas [22].

Since 2011, the Egyptian government has struggled to reconcile economic reform with social justice. Mansour argues that while recent reforms aimed at economic stabilization, they often came at the expense of equity and accessibility. Although the government adopted the rhetoric of social justice, policies like subsidy removal, tax hikes, and infrastructure spending have disproportionately affected lower- and middle-income groups. The broader neoliberal restructuring, through privatization and reduced public spending, has left vulnerable populations with unequal access to essential services [23].

Several legislative measures in Egypt, such as the Health Insurance Law (UHI) and various subsidy programs, aim to reduce financial burdens and improve healthcare affordability for low-income groups. However, these laws often lack effective enforcement, and gaps in the legal-institutional framework continue to leave vulnerable populations exposed to significant out-of-pocket costs and limited service access [24].

Addressing healthcare inequities and enhancing service quality are critical to achieving national health goals. Current disparities in healthcare access and resource allocation disproportionately affect vulnerable populations. This indicates the need for comprehensive health policies that balance efficiency with equitable access [25]. In response, Egypt enacted the Universal Health Insurance law to establish a more inclusive financing system and reduce household healthcare expenditures. While this is a major step toward equity in healthcare, its impact will depend on overcoming logistical, financial, and institutional challenges within the existing health system [26].

Egypt’s healthcare reform faces complex challenges. Service quality, accessibility, and system efficiency remain major concerns. Despite reform efforts, the sector still suffers from limited public funding, high out-of-pocket costs, and poor infrastructure, especially in rural areas. Recent initiatives include financing reforms, decentralization, and greater private sector involvement. These reforms are presented by policymakers as mechanisms to make the system more responsive and efficient. However, without strong public investment and equity-focused policies, reforms may deepen existing inequalities. A balanced approach is needed to ensure all socioeconomic groups benefit [27, 28].

Privatization, driven by neoliberal reforms, is often presented in policy analyses as offering opportunities for improved service quality, but also as posing risks of exacerbating inequalities in access, particularly for low-income populations [29]. Corporatizing public hospitals as a means of improving efficiency and service quality within Egypt’s healthcare sector involves transforming hospitals into semi-autonomous entities with management structures similar to private corporations, which could introduce accountability and performance-based management. However, this shift risks prioritizing profitability over equitable access to healthcare, particularly for low-income populations who rely on public health services. Indicating the need for careful policy design to prevent exacerbating healthcare inequalities [30, 31]. While corporatization retains public ownership, it has been widely understood as part of a reform trajectory that can facilitate subsequent privatization under conditions of weak regulatory oversight. The recent enactment of the 2024 Law on Private Sector Management of Public Healthcare Facilities marks a continuation of this trend, prompting questions about its potential implications for health equity and the sustainability of public health objectives.

#### Structure and framing of Law 87/2024

The following description draws directly on the official text of Law No. 87 of 2024 as published by the Egyptian government. The 2024 Egyptian Law No. 87 represents a critical shift in the country’s healthcare policy, granting the private sector an active role in the management, operation, and development of public healthcare facilities. Under this law, private entities, including both Egyptian and foreign investors, can enter into concession contracts with the Egyptian government for periods ranging from three to fifteen years. These contracts allow the private sector to manage and upgrade public healthcare facilities, with the intention of enhancing service quality and operational efficiency through private investment and expertise.

Several stipulations have been incorporated to address public health concerns and protect existing public sector interests. Notably, the law mandates that a portion of healthcare services provided under private management must remain accessible to public healthcare beneficiaries, ensuring that vulnerable populations retain access to essential services. Additionally, the legislation requires that at least 25% of the existing workforce in these facilities be retained, aiming to mitigate job loss concerns and maintain institutional knowledge within the public healthcare system.

To address potential crises, the law also specifies that healthcare facilities managed by the private sector must be prepared to offer essential services during public health emergencies, ensuring that privatization does not compromise the availability of critical care. Executive regulations accompanying the law provide further details on implementation, including compliance with national healthcare standards and adherence to operational guidelines. This legislation signifies a strategic effort to harness private investment to support and potentially elevate Egypt’s public healthcare infrastructure, but it also raises questions about the long-term impact on equity, accessibility, and the balance between public and private sector interests in healthcare.

### Findings from the round table discussion

The findings from the roundtable discussion are organized into four main sections, each corresponding to one of the research questions. Within each section, key themes that emerged from the discussion are presented.

#### The neoliberal foundations of Egypt’s Law 87

##### Theme 1: Historical roots of neoliberal reforms

Several experts traced Egypt’s neoliberal transformation back to the Infitah (open-door) policy in the 1970s. Participant 2 detailed how the USAID, World Bank, and IMF embedded neoliberal logic into Egypt’s health policy through decades of aid-driven reform projects, such as: The FLASK Project (1977–1987) for infrastructure mapping, the Cost Recovery Program (1987–1997) introducing patient fees, and the Health Reform Project (post-1997) pushing for privatization and restructuring. He emphasized that these projects reshaped the state’s role and introduced concepts like contract-based employment and private sector expansion into health governance.

All three programs promoted a shift toward neoliberal restructuring of the health system, clearly visible in their titles like ‘cost recovery’ and ‘health reform.’ Participant 2

Taken together, these aid-driven reform projects illustrate how Egypt’s neoliberal transformation emerged through an interaction between external financial and technical influence and domestic political economy dynamics, shaped by fiscal constraints and the institutional configuration of the health sector.

##### Theme 2: Neoliberalism as ideology, not just policy

Experts like Participant 11 and 6 highlighted that neoliberalism is more than a set of economic reforms; it represents a hegemonic paradigm. It frames health as an individual responsibility, commodifies care, and assumes market efficiency in inherently non-market systems.

Health is a textbook case of market failure… applying neoliberal logic to healthcare is problematic. Participant 11

Participant 6 strongly criticized the underlying ideological foundations of the law, arguing that it promotes austerity, privatization, and foreign investment in core public services, all of which are rooted in decades of neoliberal dominance in Egypt.

This law is rooted in neoliberalism… in a country where more than a third live in poverty, and the middle class is shrinking. Participant 6

##### Theme 3: Policy continuity amidst ideological incoherence

While some experts acknowledged that private sector involvement in healthcare may be inevitable, many emphasized that Law No. 87 is not an isolated development but rather a continuation of Egypt’s long-standing neoliberal reform trajectory. However, concerns were raised about the lack of a coherent policy direction. As Participant 3 noted, Egypt is not operating under a fully realized neoliberal model but rather navigating a system marked by ideological incoherence, a mix of market-oriented reforms layered over weak public institutions and unresolved commitments to state-led service provision:

Egypt’s system isn’t purely neoliberal, it reflects a mix of conflicting paradigms, where privatization efforts are layered over a weak public sector with lingering welfare expectations. Participant 3

This incoherent governance structure reflects the legacy of partially implemented neoliberal reforms, which have produced contradictory policy frameworks, limited regulatory capacity, and inconsistencies in service delivery.

##### Theme 4: Globalization and external conditionalities

Experts like Participant 10 and 7 drew connections between Egypt’s neoliberal trajectory and global institutions like the WTO and TRIPS agreement, which treated essential medicines as commodities and shaped Egypt’s policy choices through external conditionalities.

TRIPS made drugs commodities, legally equivalent to mobile phones. Participant 10

This global context normalized privatization, deregulation, and market-based health governance, directly influencing the framing of Law 87.

##### Theme 5: Erosion of the rights-based approach

Several speakers noted a discursive shift from health as a human right to health as a transactional service. This shift, they argued, reflects the broader impact of neoliberalism on social policy and explains why laws like 87 prioritize efficiency and investment over equity and public accountability.

We no longer speak of health as a right. People just ask: ‘What can I afford?’ Participant 10

#### Economic and health system challenges driving law 87

##### Theme 1: Economic pressures and fiscal constraints

Multiple experts pointed to rising economic strain on the state, especially in fulfilling its obligations under the Universal Health Insurance Law. Experts 1 and 11 emphasized the “upward pressure” on public finance, including: inability of the state to pay insurance premiums on behalf of the poor, fragmented financing across multiple agencies (MOHP, UHIA, university hospitals), delays in Universal Health Insurance (UHI) rollout due to limited fiscal space.

Government spending couldn’t keep up with the increasing needs… This fragmentation in financing pushed policymakers to consider private sector alternatives. Participant 11

These constraints reflect deeper structural limits on state capacity, including chronic fiscal austerity, fragmented institutional arrangements, and the cumulative weakening of public financing and regulatory functions under successive reform programs.

##### Theme 2: State investment, system failure, and the case for privatization

Participant 1 described how state investment has focused heavily on physical infrastructure, often resulting in newly built hospitals equipped with modern facilities but lacking the necessary human resources and operational systems to function effectively.

I visited hospitals built by the state, beautiful facilities full of equipment, but not a single doctor in sight. Participant 1

Such patterns are consistent with aid-influenced investment models, in which capital-intensive projects prioritize visible infrastructure while recurrent investments in staffing, operations, and system governance remain constrained. In this context, private sector management is seen by some as a strategy to activate and optimize underutilized infrastructure, although concerns remain about equity and sustainability.

##### Theme 3: Workforce shortages and migration

The law also emerges against a backdrop of severe health workforce shortages: Physicians and nurses are migrating at high rates. Existing professionals are demotivated by low pay and poor conditions.

Participant 5 and 2 both warned that Law 87 may exacerbate these issues by failing to guarantee workforce retention or training, and even allowing foreign hiring quotas, which may further discourage Egyptian professionals.

We already face severe shortages in doctors and nurses… Who guarantees staff retention under the new model? Participant 5

Doctors in Egypt earn the lowest wages among all African countries… This law may accelerate the brain drain. Participant 2

##### Theme 4: Stalled universal health insurance implementation

Participant 9 and 8 linked the law’s emergence to the slow and uneven implementation of the UHI system, where many governorates remain uncovered and coordination between UHI institutions is weak.

We need to analyse how Law 87 interacts with the UHI law. Where is the overlap, and what kind of conflict or redundancy might emerge? Participant 9

They raised concerns that the state, unable to fully roll out the UHI, may have resorted to private management as a substitute for direct public investment in operational capacity and service delivery, one that risks fragmentation if not integrated with existing reforms.

##### Theme 5: Shift toward a rentier state logic

Participant 10 described what they perceived as rent-seeking dynamics within health sector governance, in which public health infrastructure is increasingly treated as a revenue-generating asset under fiscal constraint; such arrangements may create incentives for private operators to prioritize commercially viable services, raising concerns about equity and access.

It’s in the state’s interest to lease out health facilities to generate income… but this comes at the expense of equity and access. Participant 10

This reorientation of state priorities creates fertile ground for laws like 87, which encourage BOT (Build-Operate-Transfer) models as tools for private investment.

#### Implications of Law 87 for health equity and access

Several of the equity and access concerns identified by experts can be directly traced to specific provisions of Law No. 87. The authorization of long-term concession agreements for private management of public facilities introduces incentives that prioritize financial viability, which international and regional health policy literature has shown can disadvantage low-income populations and rural areas in the absence of strong regulatory enforcement. Similarly, the law’s requirement to retain only a portion of the existing public workforce, combined with increased managerial discretion over staffing, aligns with patterns observed in market-oriented health reforms where workforce segmentation and internal inequities tend to widen over time. Finally, while the law includes general obligations to maintain services for public beneficiaries, comparable experiences from health system reforms in similarly positioned economies suggest that such provisions often depend heavily on contract design and state oversight capacity, without which equity safeguards may remain largely nominal. Together, these linkages help explain why the law’s structural design raises concerns about access, equity, and system cohesion, even in the absence of explicit exclusionary intent.

##### Theme 1: Potential Benefits: Efficiency, Quality, and Infrastructure Expansion

Several experts, including participants 13 and 8, acknowledged potential benefits of private sector involvement: Improved efficiency in hospital management, Cost-effectiveness due to more structured service delivery, Increased investment through incentives like land-for-hospital deals, and administrative clarity via a “single-window” investment approach.

Operation by the private sector could lead to better quality, improved efficiency, and reduced administrative burdens, if implemented carefully. Participant 8

The law introduces a clear investment path for health infrastructure, similar to what we have in utilities… This is a legal and administrative step forward. Participant 13

##### Theme 2: Risks, commodification and inaccessibility

However, these perceived benefits were heavily contested. A majority of experts warned that Law 87 could deepen inequities, especially in: access to affordable services, out-of-pocket spending, particularly for the poor, fragmentation of service provision, especially where the UHI is not fully active.

Private operators will seek profit, which could lead to price increases, this will hit the poor, who are the primary users of public hospitals. Participant 8

Already, 62% of health spending is out-of-pocket. What happens when more facilities are handed to private operators? Participant 6

##### Theme 3: Weak regulatory oversight

Experts repeatedly raised concern over Egypt’s limited regulatory and governance capacity. While the law includes provisions for oversight, participants expressed doubt that enforcement would be adequate.

We have a long history of absent state oversight… regulation exists on paper but not in practice. Participant 6

What matters most is not the law, but how it is implemented. Without strong governance, equity will suffer. Participant 5

The lack of an independent regulatory body and weak integration with existing UHI oversight mechanisms were identified as major risks.

##### Theme 4: Threats to the health workforce

Experts also discussed the destabilizing effect on the public health workforce. Particularly: reassigning or displacing 75% of staff, allowing up to 15% foreign hires, undermining morale and retention in an already strained workforce.

In a country facing health workforce migration, we’re allowing 15% foreign hiring while our doctors flee? That’s dangerous. Participant 6

Doctors are leaving by the plane-load… this law could make things worse. Participant 2

##### Theme 5: Loss of health as a public good

A more philosophical concern was raised by Participant 10, 7 and others: the law signifies a shift away from viewing health as a public good or human right, and toward treating it as a commodity.

We no longer speak of health as a right. People ask: ‘What can I afford?’ not ‘What do I deserve?’ Participant 10

If equity and access aren’t at the core, this isn’t reform, it’s regression. Participant 7

#### Strategies to mitigate the potential adverse effects of Law 87

##### Theme 1: Limit implementation to UHI-covered governorates

A key strategy proposed by participant 8 was to restrict the implementation of Law 87 to governorates already covered under Phases 1 and 2 of the Universal Health Insurance System (UHIS). This would ensure that a financing and purchasing mechanism is in place to protect the poor.

The second component, operating public hospitals, should be limited to governorates already under the UHI system. This way, the underprivileged are protected from out-of-pocket payments. Participant 8

This was echoed by participant 9 and others, who warned that implementation in areas without active UHI coverage could lead to chaos, inequality, and unregulated pricing.

##### Theme 2: Strengthen primary healthcare and referral systems

Several participants stressed that reinvesting in primary care is essential to reducing costs and improving access. Participant 1 emphasized that neglecting primary care undermines the entire system and overloads hospitals.

We must invest in primary healthcare to ease the burden on hospitals. Many facilities are sitting empty. We should activate them rather than bypass them. Participant 1

We’re all trying to fix secondary and tertiary care while ignoring our biggest asset: primary healthcare. Participant 1

##### Theme 3: Independent oversight and governance reform

There was a consensus on the urgent need for a strong, independent regulatory body to monitor contract terms, enforce service quality standards, and protect equity. Participants 5, 11, and 13 emphasized that without independent oversight, private sector engagement poses major risks.

We need a strong and independent oversight body, clear regulatory guidelines, and continuous evaluation. Participant 5

The legal and governance frameworks to regulate the private sector are currently lacking. That must be addressed urgently. Participant 11

##### Theme 4: Integration with UHI and avoiding legal fragmentation

Participant 11 warned of legal inconsistency between Law 87, the UHI Law, and the Public-Private Partnership Law (No. 67 of 2010). Experts urged the harmonization of legal frameworks to avoid duplication, conflict, and inefficiency.

Law 87 doesn’t reference the existing PPP Law at all. We need alignment between legal structures to avoid implementation chaos. Participant 11

##### Theme 5: Equity-oriented contract design and public accountability

Experts proposed including equity safeguards in service contracts, such as: mandated service quotas for low-income patients, price caps on essential services, transparent performance reporting, and worker protection clauses.

We must set criteria that guarantee affordability, access, and retention of skilled professionals. Participant 6

Participant 13 added that pricing and service coverage should be negotiated through public institutions, not left to private discretion.

##### Theme 6: Civil society engagement and citizen voice

Participant 7 and others emphasized the need for citizen participation, civil society oversight, and democratic accountability in all stages of law formulation, implementation and reform monitoring.

Unless we take citizens’ input seriously and I mean structurally, not symbolically, we’ll repeat this cycle of failed reforms. Participant 7

Policies must reflect real societal needs, not just economic imperatives. Participant 12

### Triangulation of data sources

Triangulation of the roundtable findings with the analysis of Law 87, its executive regulations, and the supporting literature showed areas of both convergence and divergence. All sources identified fiscal pressures, workforce shortages, and governance gaps as key contextual drivers of the law. While the legal text and literature predominantly frame the reform as a mechanism to improve efficiency and attract investment, experts highlighted concerns related to equity, regulatory oversight, and the implications for the health workforce that are not explicitly addressed in the law. This triangulation enhanced the credibility of the analysis by demonstrating where the legal provisions and broader evidence base aligned with, or diverged from, expert interpretations grounded in practical experience within Egypt’s health system.

## Discussion

This study examined Egypt’s Law No. 87 of 2024 through the lens of political economy, health equity, and governance. While the law reflects elements of neoliberal reform, particularly in its emphasis on private sector involvement, investment, and efficiency, it is not solely a product of neoliberal ideology. Rather, it emerged from a complex mix of external economic pressures, institutional limitations, rising healthcare demand, and long-standing inefficiencies in public service delivery. External actors such as international financial institutions have influenced reform directions, but domestic factors such as fiscal constraints, demographic pressures, and persistent gaps in public service quality have played an equally central role. Expert perspectives revealed how these forces have played out on the ground, especially in terms of workforce shortages, deteriorating infrastructure, and rising reliance on private care. From a political economy perspective, the law signals a redistribution of roles between public and private actors, building on a policy trajectory that has prioritized cost-effectiveness and administrative reform, often without fully addressing underlying equity concerns [7]. These trends raise serious questions about whether such reforms, in the absence of strong governance and legal integration, can improve access and outcomes for vulnerable populations [16, 20].

The analysis suggests that neoliberal political economy dynamics help explain the direction and design of Law 87, particularly its reliance on private management and investment logic. However, the anticipated risks identified in this study stem not only from ideological orientation, but also from longstanding institutional and regulatory constraints within Egypt’s health system. Market-oriented reforms can produce diverse outcomes depending on the strength of oversight, contract enforcement, and governance capacity. In contexts where administrative institutions are fragmented or under-resourced, implementation risks become magnified. Thus, the potential challenges associated with Law 87 reflect an interaction between reform design and institutional capacity, rather than being attributable to a single explanatory factor.

### Neoliberal foundations of Law 87

Egypt’s trajectory toward neoliberal health reform has been shaped by external pressures, especially from the World Bank and the International Monetary Fund (IMF). These institutions, rooted in global neoliberal ideology, have long influenced public sector reforms in low- and middle-income countries [32]. In Egypt, they acted as tools of economic and policy pressure. Today’s economic crisis and rising debt have made Egypt more vulnerable to these pressures. There is growing pressure to reduce public spending and open new spaces for private sector investment. This shift did not affect everyone equally. Reforms opened new opportunities for elites and politically connected actors. They gained access to key sectors, including real estate, private hospitals, and health-related investments. Meanwhile, the burden shifted to ordinary people, and expectations changed. People no longer demand health as a right. They ask what they can afford. This shift is not only economic, it is ideological. It reflects how decades of neoliberal thinking reshaped the meaning of care, from public good to personal responsibility.

However, Egypt’s policy path has never been fully neoliberal. The country carries a legacy of the socialist era [33]. It also has a long tradition of rights-based social movements and intellectual voices that support the idea of health as a public good. These forces have challenged the full adoption of neoliberal reforms and helped preserve some state-led approaches. As one expert noted, Egypt has never been completely neoliberal. Instead, it has followed a more uneven path, where neoliberal reforms are layered over a system still influenced by welfare-state ideals.

This has produced a mixed health system, where public and private actors coexist, but often in parallel rather than in coordinated partnership. Despite common discourse, Egypt did not formally privatize its public health assets prior to Law 87. The private sector grew in response to unmet demand and the deterioration of public services, not through state divestment. Law 87 represents the first legal framework that explicitly invites private management into public healthcare facilities, marking a shift in the policy landscape.

Moreover, successive governments have continued to implement large-scale health and social programs, such as the Takaful and Karama cash transfer program [34] and the 100 Million Seha initiative [35], that reflect state responsibility for health and welfare, even as market-oriented reforms advance. This illustrates that Egypt’s trajectory is shaped as much by institutional path dependency and developmental imperatives as by neoliberal economic logic.

This created a mixed system. Neoliberal reforms were introduced, but older welfare-state elements were never ignored. As a result, market-based policies now operate alongside state-led structures, often in conflict with one another.

### Economic and health system challenges driving Law 87

Law 87 emerged in the context of a weak public health system. Gaps in quality, under-resourced facilities, and poor outreach, especially in rural areas, have long undermined public trust [36, 37]. Egypt is also facing major demographic shifts. Population growth and an aging population are increasing the burden on the Ministry of Health and the state. From 1960 to 2023, Egypt’s population increased from 26.6 million to 114.5 million, representing a growth of over 330% in 63 years [38]. The number of Egyptians aged 60 and over is projected to more than double from 8.4 million (8% of the population) in 2020 to 22 million (14%) by 2050 [39]. At the same time, the government is focused on economic growth. While recent health initiatives, including the Universal Health Insurance Law, reflect reform efforts, the broader policy direction remains shaped by economic priorities.

One expert noted that health spending is not guided by deep analysis or a clear national strategy. These points to weaknesses in planning, policy choice, and health system governance. These gaps are especially visible in workforce policy. Egypt’s public health system is already experiencing sustained out-migration of doctors and nurses, driven by low wages, poor working conditions, and limited career progression [40]. From a political-economy perspective, health workforce dynamics in Egypt are not only technical issues of staffing but are also shaped by labour relations, professional hierarchies, and the historical role of medical syndicates and professional associations in negotiating employment conditions. Law 87 intervenes in this sensitive terrain by allowing private operators substantial discretion over staffing decisions, requiring only partial retention of the existing workforce. In the absence of strong labour protections and regulatory oversight, this flexibility may weaken collective bargaining power, exacerbate segmentation between public and privately managed facilities, and further intensify existing inequalities within the health workforce. These dynamics are critical not only for workforce stability but also for the law’s long-term implications for service continuity, equity, and public sector capacity.

Finally, Egypt’s economic crisis has intensified the push to reduce public spending [41]. Experts raised concern that the state may be moving toward a rentier approach, leasing public assets to generate revenue. In this view, the law reflects an easy short-term solution rather than a long-term strategy for health system reform. These conditions created fertile ground for reforms that shifted responsibility to the private sector while easing fiscal pressure on the state, a common strategy in neoliberal health governance. However, it is essential to recognize that these choices were not made solely out of ideological commitment. They also stem from structural limitations, growing population needs, and the absence of sufficient fiscal space to expand public provision alone.

### Implications of Law 87 for health equity and access

Law 87 assumes that involving the private sector will enhance efficiency and improve quality of care. While private management may streamline operations, longstanding health policy scholarship cautions against reducing healthcare to a purely business model. Healthcare is widely recognized as a public good with social and ethical dimensions that extend beyond market efficiency. In systems shaped predominantly by market logic, access can become stratified, with service quality and timeliness varying according to ability to pay.

The law introduces risks to both equity and accessibility. Private investors are unlikely to prioritize rural areas or low-profit services. It is important to clarify that profitability under Law 87 would not necessarily depend on high state reimbursement rates alone. Private operators may generate returns through a combination of efficiency gains, selective service mix, differential pricing of non-covered services, and cross-subsidization between publicly financed and privately paying patients. In the absence of clearly defined contractual safeguards and transparent pricing structures, such mechanisms could alter service priorities over time. The financial and equity implications of the reform will therefore depend significantly on contract design, reimbursement rules, and the strength of regulatory oversight.

If these governance mechanisms are weak or inconsistently enforced, service provision may gradually shift toward commercially viable segments, potentially leading to the marginalization of underserved populations. In addition, an increased emphasis on personal financial responsibility for care may weaken the practical realization of health as a right. When public services are managed for profit, access may become more conditional than guaranteed, particularly for vulnerable groups.

At the same time, it is important to acknowledge that many Egyptians already rely heavily on private providers, not necessarily because they reject public care ideologically, but due to gaps in availability, convenience, and perceived quality. Law 87 formalizes an already existing reliance. Its ultimate impact will depend on regulatory strength, inclusion criteria, and whether equity safeguards are effectively embedded in contractual arrangements.

### Law 87 in relation to the universal health insurance and PPP Laws

Law 87 cannot be analysed in isolation. Its implementation is closely tied to other major health and legal reforms, especially the Universal Health Insurance Law and the Public-Private Partnership (PPP) Law No. 67 of 2010. However, there is no clear explanation of how these laws will interact. The UHI law aims to provide universal coverage and reduce out-of-pocket spending. It follows a social protection model with a focus on equity and public responsibility [42]. In contrast, Law 87 introduces market logic into the management of public hospitals. The PPP law sits somewhere in between, promoting collaboration but requiring strong regulation.

Without clear legal and institutional alignment, these laws risk working against each other. There is no mechanism to ensure that services provided under Law 87 will be covered by UHI or accessible to its beneficiaries. There is also no clarity on which law takes precedence when private providers are contracted. These gaps raise questions about legal coherence and policy consistency.

More importantly, the three laws reflect competing visions of health policy. The UHI law is rooted in a rights-based, universalist model. Law 87 is grounded in neoliberal assumptions about efficiency and investment. The PPP law depends on strong governance and clear contracts. Without coordination, the system may become more fragmented, less transparent, and harder to regulate. This makes the case for a unified policy and legal framework that protects both public health goals and financial sustainability. The lack of legal alignment also reflects a governance gap, where fragmented institutional mandates weaken coherence and undermine policy goals.

### Governance as a cross-cutting challenge

Across all of these issues lies the question of governance. Law 87 has been passed, but its governance mechanisms remain unclear. Key elements like accountability, transparency, and oversight are not well defined. Yet these are essential for ensuring that the law supports equity and does not worsen disparities. Good governance is not a technical detail; it is the foundation for public trust and effective implementation.

The law also raises concerns about shifting authority from public institutions to private actors without clear checks and balances. Traditional stewardship roles of the state may be weakened if core decisions on service delivery, workforce, and pricing are delegated to private operators. Furthermore, the law was developed without broad stakeholder consultation, limiting public voice in shaping health policy. These gaps challenge the legitimacy and sustainability of the reform.

The PPP Law could be used as a supporting framework, but only if applied with strong regulation and institutional coordination. Issuing a law is the easiest step. The real challenge lies in how it is implemented, monitored, and by whom. Without clear roles, strong institutions, and adequate resources, the risk of failure is high. Law 87 could end up like other reforms that were not centred on people’s needs and did not deliver on their promises. The question is not only what the law says, but whether the system can govern it in a way that protects public health.

## Conclusion

This study examined Egypt’s Law No. 87 of 2024 through the lens of political economy, governance, and health equity. The findings suggest that the law reflects a progression in Egypt’s health policy, driven by both economic pressures and domestic challenges, including resource limitations and the growing demand for services. While the law aligns with market-oriented approaches, it is not purely ideological. It responds to a complex policy environment shaped by fiscal constraints and performance concerns in the public sector.

The first concern relates to governance and legal coherence. Law 87 was introduced alongside existing frameworks like the Universal Health Insurance (UHI) Law and the Public-Private Partnership (PPP) Law, but with no clear explanation of how these laws will interact. This lack of alignment creates confusion and raises concerns about duplication, legal gaps, and regulatory inconsistency. For reforms to succeed, implementation must be anchored in a unified legal and institutional framework, with strong coordination and oversight.

The second issue is equity. Without clear safeguards, there is a real risk that the law could deepen existing disparities. Private operators may avoid low-profit or hard-to-reach areas, leaving vulnerable populations behind. Equity cannot be assumed; it must be built into the law through enforceable mechanisms such as service mandates for underserved areas, price regulations, and workforce protections.

The third point concerns the private sector itself. Law 87 formalizes a role the private sector has already played informally, but it does so without yet defining clear boundaries or obligations. If properly regulated, this may offer opportunities to improve service efficiency and relieve pressure on public resources. But if left unchecked, it could entrench a dual-track system and weaken public sector capacity.

The law’s future impact will depend on how it is implemented. Capable institutions, clear accountability mechanisms, and genuine public oversight will be critical. Without them, the law may widen gaps in the health system rather than improve access and outcomes.

## Data Availability

No datasets are available for public access. The roundtable transcript is confidential and cannot be shared.

## Abbreviations

IMF: International Monetary Fund
IRB: Institutional Review Board
PPP: Public-Private Partnership
UHI: Universal Health Insurance
UHIS: Universal Health Insurance System
WHO: World Health Organization
